# Long-term outcomes of the 2-week schedule of hypofractionated radiotherapy for recurrent hepatocellular carcinoma

**DOI:** 10.1186/s12885-018-4953-x

**Published:** 2018-10-26

**Authors:** Jongmoo Park, Jinhong Jung, Daegeun Kim, In-Hye Jung, Jin-hong Park, Jong Hoon Kim, Sang-wook Lee, Sang Min Yoon

**Affiliations:** 1 Department of Radiation Oncology, Asan Medical Center, University of Ulsan College of Medicine, 88, Olympic-ro 43-gil, Songpa-gu, Seoul, 05505 Republic of Korea; 2 Department of Radiation Oncology, Chungbuk National University Hospital, Cheongju, 28644 Republic of Korea; 3 University of Ulsan College of Medicine, 88, Olympic-ro 43-gil, Songpa-gu, Seoul, 05505 Republic of Korea

**Keywords:** Hepatocellular carcinoma, Hypofractionated radiotherapy, Salvage therapy

## Abstract

**Background:**

The 2-week schedule of hypofractionated radiotherapy as a salvage treatment for hepatocellular carcinoma (HCC) has previously exhibited promising results; this study aimed to assess its long-term clinical outcomes in patients with recurrent HCC ineligible for curative treatments.

**Methods:**

We retrospectively enrolled 77 patients (84 lesions) with HCC who were treated with hypofractionated radiotherapy between December 2008 and July 2013. Primary inclusion criteria were HCC unsuitable for curative treatments and HCC located within 2 cm of a critical normal organ. We administered 3.5–5 Gy/fraction for 2 weeks, resulting in a total dose of 35–50 Gy.

**Results:**

The median follow-up period was 33.6 (range, 4.8–78.3) months. The 3- and 5-year overall survival rates were 52.3% and 40.9%, respectively, and local control rates were 79.5% and 72.6% in all treated lesions, respectively. The 5-year local control rate was better in the higher radiation dose group than in the lower radiation dose group (50 Gy: 79.7% vs. < 50 Gy: 66.1%); however, the difference was not statistically significant (*P* = 0.493). We observed grade ≥ 3 hepatic toxicity in 2 (2.6%) patients and grade 3 gastrointestinal bleeding in 1 (1.3%) patient. However, grade ≥ 4 toxicity was not observed after hypofractionated radiotherapy.

**Conclusions:**

The 2-week schedule of hypofractionated radiotherapy for recurrent HCC exhibited good local control and acceptable treatment-related toxicity during the long-term follow-up period. Thus, this fractionation schedule can be a potential salvage treatment option for recurrent HCC, particularly for tumors located close to a radiosensitive gastrointestinal organ.

**Electronic supplementary material:**

The online version of this article (10.1186/s12885-018-4953-x) contains supplementary material, which is available to authorized users.

## Background

Liver cancer is the second leading cause of cancer-related death worldwide [1], and hepatocellular carcinoma (HCC) is the most common histological type of primary liver cancer [2]. Although surgical approaches and percutaneous ablation therapies are recommended as curative treatment options for HCC, most patients with HCC are not suitable for these curative treatments. Typically, < 30% of patients undergo surgical treatment, owing to the extent of tumors, severity of underlying liver dysfunction, or limited resources. In addition, percutaneous ablation therapies are limited due to the location and/or size of HCC [3–6].

With remarkable recent technological advancements in radiotherapy, including four-dimensional (4D) computed tomography (CT), image-guided radiotherapy (IGRT), and respiratory-gated delivery, stereotactic body radiation therapy (SBRT) has emerged as an alternative treatment option for patients ineligible for the curative treatments [7–14]. A recent retrospective study demonstrated the potential role of SBRT for HCC by comparing the outcomes of radiofrequency ablation (RFA) and SBRT [15]. Despite the growing role of this high-dose radiotherapy, the use of SBRT for HCC located adjacent to radiosensitive gastrointestinal organs, such as the stomach, duodenum, esophagus, and/or large bowel, remains challenging because a high prescribed dose with a large fraction size can induce severe bleeding or perforation [11–13, 16]. Furthermore, the majority of patients with HCC present with underlying liver cirrhosis, portal hypertension, and coagulopathy, all of which exacerbate the risk of gastrointestinal toxicity in comparison to patients without chronic liver diseases.

Thus, the fractionation scheme can be modified to reduce the probability of gastrointestinal toxicities. In our previous study, we adopted a 2-week schedule of hypofractionated radiotherapy to decrease the fraction size and reported the feasibility of this scheme for recurrent HCC located adjacent to critical organs [17]. The present study aimed to assess the long-term efficacy and safety of hypofractionated radiotherapy in patients with small recurrent HCC.

## Methods

### Patient selection

In this study, we retrospectively reviewed patients who underwent the 2-week schedule of hypofractionated radiotherapy for recurrent HCC at Asan Medical Center between December 2008 and July 2013. The detailed inclusion criteria for the use of hypofractionated radiotherapy has been described in our previous study [17]. The primary indication of this treatment scheme is the location of HCC within 2 cm of radiosensitive organs such as the stomach, duodenum, esophagus, or large bowel. HCC was diagnosed on the basis of histological confirmation and/or imaging criteria of the American Association for the Study of Liver Disease. This retrospective study was approved by the Institutional Review Board of the Asan Medical Center and the requirement for informed consent was waived because of the retrospective nature of this study.

### Radiotherapy

All patients were immobilized in the supine position using a vacuum cushion. Then, each patient underwent 4D CT simulation with free breathing using a 16-slice CT (GE LightSpeed RT 16; GE Healthcare, Waukesha, WI, USA). The breathing pattern was assessed using the Real-time Position Management System (Varian Medical System, Palo Alto, CA, USA). The CT slice thickness was set to 2.5 mm. An intravenous contrast agent was used to improve the accuracy of target volume delineation. 4D imaging software (Advantage 4D; GE Healthcare) was used to sort 4D-CT datasets from 10-phase bins corresponding to the respiratory phase. One week before the CT simulation, three gold fiducial markers (CIVICO Medical Solution, Kalona, IA, USA) were implanted into the liver parenchyma under ultrasound guidance, except for those with surgical clips or compact iodized oil that could be used as a fiducial marker on cone-beam CT and fluoroscopy [18].

The gross tumor volume (GTV) was defined as an arterial enhancing lesion with washout on diagnostic dynamic enhanced CT and/or magnetic resonance imaging (MRI) at the end-expiratory phase of simulation CT imaging. The internal target volume (ITV) was defined as the summation of individual GTVs in the gated respiratory interval (mostly 30–70%) or in an entire respiratory cycle. The planning target volume (PTV) was obtained by expanding 0.5 cm from the ITV in all directions. The normal liver, esophagus, stomach duodenum, colon, kidneys, and spinal cord were contoured for dose constraint for organs at risk.

Three-dimensional conformal radiotherapy technique was used to determine radiation ports using a planning system (Eclipse; Varian Medical Systems) and the most actual beam delivery was performed with a respiratory-gated delivery technique. Of note, the total dose to the PTV was 35–50 Gy, administered in 10 fractions (5 fractions/week) and the fraction size was determined according to the maximum dose in adjacent radiosensitive organs. Over 95% of the PTV received 100% of the prescription dose and the chosen isodose covering PTV was between 85 and 90%, which was normalized to the center of the PTV. In this study, the dose constraints for normal organs were as follows: no more than 25% of the normal liver volume would receive over 50% of the prescribed dose; maximum dose to the colon and esophagus should not exceed 3.5 Gy/fraction; and the dose to the stomach, duodenum, and spinal cord should not exceed 2.5 Gy/fraction.

### Evaluation and statistical analysis

During hypofractionated radiotherapy, all patients were examined to assess the acute toxicity. After the completion of treatment, the medical history was obtained and physical examinations, complete blood counts, biochemical profiles, coagulation tests, tumor markers, and CT or MRI were performed at 2- or 3-month intervals. Notably, the toxicity, related to constitutional symptoms, gastrointestinal effects, and laboratory findings during and after the treatment was assessed according to the Common Terminology Criteria for Adverse Events (version 4.03). In addition, the deterioration of the Child-Pugh score (increased by ≥2) in the absence of tumor progression within 3 months after radiotherapy was evaluated [19].

The tumor response after radiotherapy was evaluated according to the Response Evaluation Criteria in Solid Tumor (RECIST, version 1.1) at 3 months after the completion of radiotherapy. Local failure was defined as the recurrence of a treated lesion, and progression was defined as any type of recurrence, including intrahepatic recurrence or extrahepatic metastasis for the progression-free survival (PFS). The overall survival (OS) and PFS were estimated from the date of the start of radiotherapy to the date of death or the last follow-up and to the date of death, tumor recurrence, or last follow-up, respectively, using the Kaplan-Meier method. Both univariate and multivariate analyses were performed using the Cox proportional hazards model to illustrate the association of variables with survival outcomes. The backward elimination Cox’s regression was used to select the principal risk factors in the multivariate model. All tests were two-sided, and *P* < 0.05 was considered statistically significant. All statistical analyses were performed using the SPSS software (version 21; IBM SPSS Statistics, Armonk, NY, USA).

## Results

### Patients and treatment

During the study period, 120 patients with recurrent HCC were treated with the hypofractionated radiotherapy. Of these, 43 patients were excluded from the analysis because of the following reasons: a history of previous radiotherapy to the liver (*n* = 22), presence of vascular invasion (*n* = 10), multiple viable HCCs outside the radiation field (*n* = 5), presence of extrahepatic metastasis (*n* = 3), and lost to follow-up just after the completion of radiotherapy (*n* = 3). The remaining 77 patients (84 lesions) fulfilled the inclusion criteria and were enrolled in this study (Table 1, Additional files 1 and 2). The median tumor size was 2.4 cm and approximately 30% of lesions were > 3 cm in size.

**Table 1:** Patient characteristics

Variables	Number of patients (%)
Sex	
Male	45 (58.4)
Female	32 (41.6)
Age (years)	
Median	62
Range	42–88
ECOG performance status	
0	60 (77.9)
1	11 (14.3)
2–3	6 (7.8)
Child-Pugh class	
A	56 (72.7)
B	21 (27.3)
Viral etiology	
Hepatitis B virus	51 (66.2)
Hepatitis C virus	14 (18.2)
Others	12 (15.6)
Alpha-fetoprotein (ng/mL)	
< 200	58 (75.3)
≥ 200	19 (24.7)
Tumor size (cm) (*n*=84)	
Median	2.4
Range	0.8–5.6
≤ 3 cm	59 (70.2)
> 3 cm	25 (29.8)

*ECOG* Eastern Cooperative Oncology Group

Before receiving hypofractionated radiotherapy, all patients had received various previous therapies, including hepatic resection, RFA, percutaneous ethanol injection, transarterial chemoembolization (TACE), or a combination of these treatments (Table 2). The median number of previous treatment sessions was 4 (range, 1–19). In addition, 50 (59.5%) lesions received a total dose of 50 Gy, and the remaining lesions were administered 40 or 35 Gy according to the maximum dose in adjacent normal organs as mentioned earlier. Four patients received radiotherapy using a volumetric-modulated arc therapy technique to address stomach and duodenal constraints. Most lesions (94%) were treated using the respiratory-gated technique during radiotherapy (Table 2).

**Table 2:** Treatment characteristics of 84 lesions

Variables	Number of patients (%)
Previous treatment (*n* = 77)	
TACE	41 (53.2)
TACE + RFA	17 (22.1)
TACE + PEI	3 (3.9)
TACE + RFA + PEI	1 (1.3)
TACE + Resection	6 (7.8)
TACE + Resection + RFA	6 (7.8)
TACE + Resection + PEI	1 (1.3)
TACE + Resection + RFA + PEI	1 (1.3)
RFA	1 (1.3)
Radiation dose	
35 Gy/10 fractions	7 (8.3)
40 Gy/10 fractions	27 (32.2)
50 Gy/10 fractions	50 (59.5)
Radiotherapy plan	
Three-dimensional conformal radiotherapy	80 (95.2)
Volumetric-modulated arc therapy	4 (4.8)
Respiratory-gated beam delivery	
Yes	79 (94.0)
No	5 (6.0)

*TACE* transarterial chemoembolization, *RFA* radiofrequency ablation, *PEI* percutaneous ethanol injection

### Radiotherapy response and survival outcomes

**Fig. 1:**
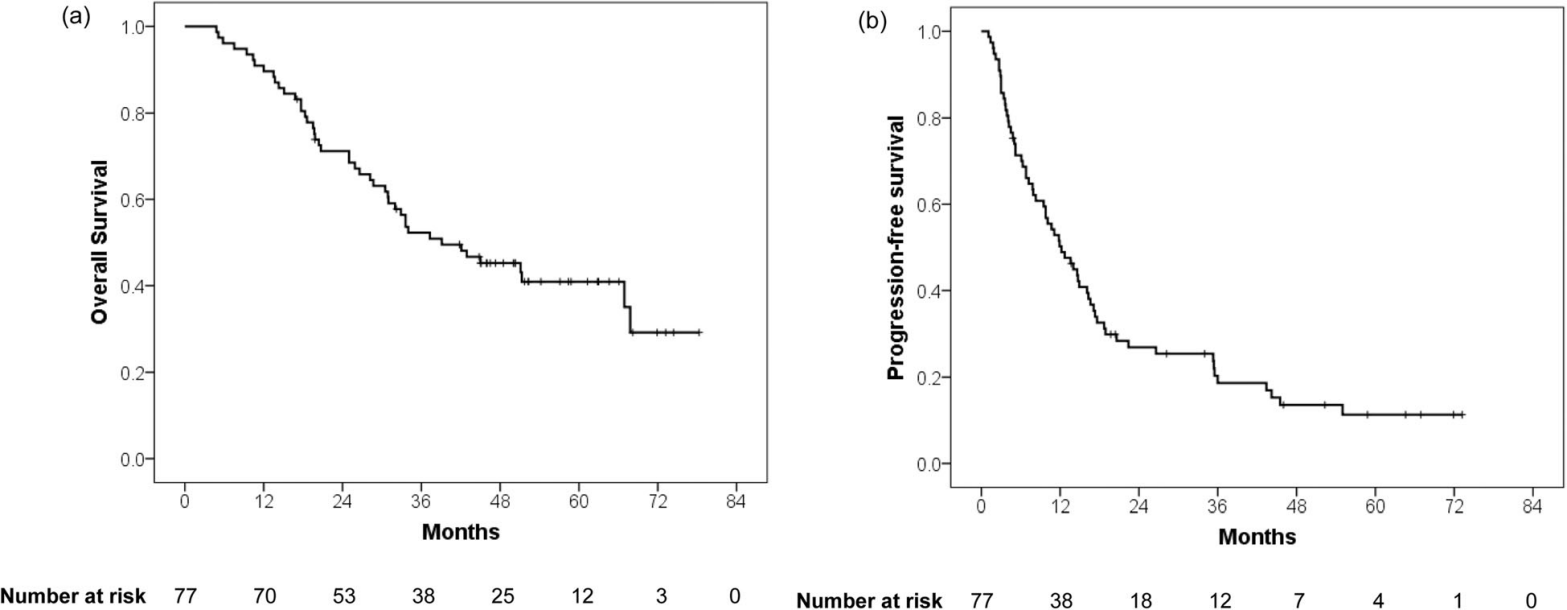
**a** The overall survival and **b** progression-free survival rates in all patients

Three months after the completion of radiotherapy, complete response, partial response, stable disease, and progressive disease were attained in 33 (39.3%), 27 (32.1%), 23 (27.4%), and 1 (1.2%) lesions, respectively. The median follow-up period for all patients and surviving patients was 33.6 (range, 4.8–78.3) and 52.0 (range, 17.1–78.3) months, respectively. At the time of analysis, 45 patients died, and 32 patients remained alive. The 3- and 5-year OS rates were 52.3% and 40.9%, respectively (Fig. 1a). In the multivariate analysis, the number of previous treatment was a significant prognostic factor for the OS (hazard ratio [HR] = 1.08; 95% confidence interval [CI], 1.01–1.16; *P* = 0.029; Table 3). Sixty-four (83.1%) patients experienced tumor recurrences after radiotherapy. The most common failure pattern was the development of a new HCC in the liver (89.1%). The 3- and 5-year PFS rates were 18.7% and 11.3%, respectively, in all patients (Fig. 1b).

**Table 3:** Factors affecting the overall survival after hypofractionated radiotherapy

Variables	Univariate analysis			Multivariate analysis		
	HR	95% CI	*P* value	HR	95% CI	*P* value
Sex	1.05	0.60–1.83	0.876			
Age	1.02	0.99–1.05	0.144	1.03	0.99–1.06	0.080
ECOG PS (0–1 vs. 2–3)	1.30	0.51–3.28	0.581			
Tumor size	1.23	0.96–1.58	0.101	1.24	0.95–1.61	0.119
Alpha-fetoprotein (log_10_)	1.24	0.92–1.67	0.154	1.17	0.86–1.59	0.323
Child-Pugh class	1.20	0.65–2.21	0.570			
Number of previous treatment sessions	1.10	1.03–1.17	0.007	1.08	1.01–1.16	0.029
Dose (50 Gy vs. < 50 Gy)	1.03	0.98–1.08	0.310			

*HR* hazard ratio, *CI* confidence interval, *ECOG* Eastern Cooperative Oncology Group, *PS* performance status

### Local control rates

**Fig. 2:**
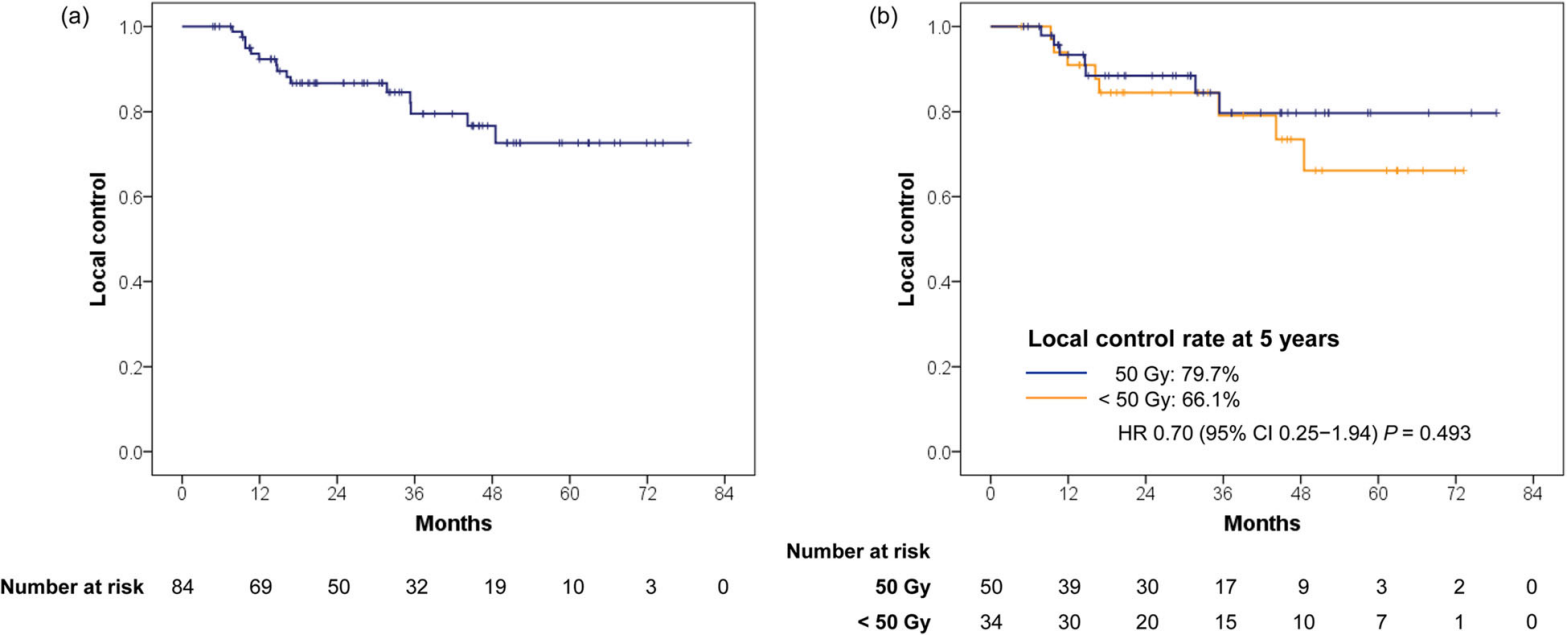
**a** The local control rate and **b** local control rates according to the prescribed dose in all lesions

The 3- and 5-year local control rates in all lesions were 79.5% and 72.6%, respectively (Fig. 2a). Based on the prescribed dose, the 5-year local control rate was better in the higher radiation dose group than the lower radiation dose group (50 Gy: 79.7% vs. < 50 Gy: 66.1%); however, the difference was not statistically significant (HR = 0.70; 95% CI, 0.25–1.94; *P* = 0.493; Fig. 2b). There was no other clinical parameter that was associated with local tumor control after hypofractionated radiotherapy (Additional file 3: Table S1).

### Treatment-related toxicity

All patients received planned radiotherapy without any interruptions. Table 4 summarizes treatment-related toxicities in this study. Although the worsening of the biochemical blood tests, such as transaminase, bilirubin, or alkaline phosphatase, was the most common toxicity, however, the incidence of grade ≥ 3 toxicity was only 2.6% (2 patients); these elevated transaminases spontaneously normalized within 3 months with supportive care only. An elevation of the Child-Pugh score ≥ 2 without tumor progression was observed in 4 (5.2%) patients. The most common acute constitutional symptoms other than hepatic toxicities were fatigue, anorexia, and nausea; however, these toxicities were mostly grade 1 and resolved immediately after radiotherapy.

**Table 4:** Treatment-related toxicity

Adverse events	Number of patients				
	Grade 1	Grade 2	Grade 3	Grade 4	Grade 5
Acute					
Fatigue	15	2	0	0	0
Anorexia	8	2	0	0	0
Abdominal Pain	5	3	0	0	0
Nausea	6	0	0	0	0
Biochemical					
Transaminase	30	3	2	0	0
Bilirubin	19	13	0	0	0
Alkaline phosphatase	16	0	0	0	0
Late					
Rib fracture	1	0	0	0	0
Biliary stricture	1	0	0	0	0
Gastrointestinal bleeding	0	0	1	0	0

During the follow-up period, one patient who was administered 50 Gy experienced grade 3 gastrointestinal bleeding after 5 months of radiotherapy. The tumor was located in segment 2, and the distance between the stomach antrum and HCC was 1.5 cm on simulation CT. Despite the maximum dose to the stomach being 30 Gy in the treatment planning, endoscopy revealed active bleeding foci in the stomach antrum. The patient received supportive care, including blood transfusion and endoscopic hemostasis, and subsequently recovered well. Regarding other late toxicities, a rib fracture (grade 1, 15 months after radiotherapy) and a biliary stricture (grade 1, 11 months after radiotherapy) developed; however, these toxicities did not require specific treatment. Of note, no patients experienced grade 4 or 5 toxicity after hypofractionated radiotherapy.

## Discussion

With the development of radiotherapy technology, SBRT (usually ≤5–6 fractions) has found utility in the management of small HCC when curative treatment modalities cannot be applied [7, 8, 10–13]. Although high-dose radiation offers the biological advantage of contributing to the local tumor control, radiation-induced toxicity is a concern in cases of HCC adjacent to radiosensitive gastrointestinal organs. Besides, the majority of patients with HCC present with an underlying chronic liver disease, which could exacerbate the risk of gastrointestinal toxicities, owing to the hypothesis of the impairment of the mucosal defense mechanism [20]. Several studies investigating SBRT using total doses of 25–60 Gy in 3–5 fractions reported grade ≥ 3 gastrointestinal toxicities, such as bleeding or perforation [11–13, 16, 21, 22]. Jang et al. reported that 5 (6%) patients whose tumors were located in the vicinity of the gastrointestinal tract experienced grade 3 or 4 gastrointestinal ulcers or perforations after 3-fraction SBRT for HCC [13]. In addition, Huertas et al. reported three (3.9%) gastric or colonic ulcers, although they used alternative SBRT fractionation protocols of 50 Gy in 4 or 5 fractions, rather than 45 Gy in 3 fractions, in case of tumors located adjacent to the gastrointestinal tract [21]. Although SBRT exhibits a promising local control in patients with HCC, a severe complication should be considered in patients with HCC adjacent to gastrointestinal organs even if the overall incidence is relatively low.

In the present study, the 2-week schedule of hypofractionated radiotherapy for recurrent HCC located adjacent to radiosensitive gastrointestinal organs yielded an encouraging long-term local control rate (72.6% at 5 years) with acceptable toxicity. Regarding the local tumor control, this fractionation scheme exhibited a marginally lower local control rate compared with that of SBRT delivering higher doses with 3–5 fractions [7, 8, 11, 12, 21]. However, the patients in our study had no effective local treatment options, such as resection, transplantation, RFA, or TACE, owing to the complicated clinical situations. Moreover, their tumors were located adjacent to the stomach, duodenum, esophagus, or colon; this could be another hindrance in deciding the optimal treatment. When SBRT using 3–5 fractions cannot be performed due to the imminent risk of gastrointestinal toxicity, this 2-week schedule radiotherapy could be safely implemented. In this study, all lesions located within 2 cm from gastrointestinal organs were treated with a smaller fractional dose (3.5–5 Gy), and only 1 (1.3%) patient experienced grade 3 gastrointestinal bleeding, which, however, recovered with conservative management. Table 5 summarizes the clinical outcomes of the recent studies of SBRT or 2-week schedule of hypofractionated radiotherapy for HCC.

**Table 5:** Summary of the recent studies on stereotactic body radiation therapy or 2-week schedule of hypofractionated radiotherapy for hepatocellular carcinoma

Author (year)	Design	No. of pts.	Clinical condition & indication			Dose prescription, median (range), Gy/fractions	LC, %	OS, %	Toxicity	
			PVTT	Tumor size, median (range), cm	CP class				Liver	GI
Andolino [9] (2011)	Phase I/II	60	NA	3.1 (1.0–6.5)	A,B	CP-A: 44/3 (30–48) CP-B: 40/5 (24–48)	90 (2Y)	67 (2Y)	CP class change: 20% (A➔B: 12%, B➔A: 8%) Hepatic failure: 7% (all with a CP score ≥ 8)	No GI toxicity ≥ G3
Kang [12] (2012)	Phase II	47	Yes (11%)	2.9 (1.3–7.8) (sum of size)	A,B7	57/3 (42–60/3)	95 (2Y)	69 (2Y)	No RILD CP class change A➔B: 13%	GI toxicity G3: 6% Gastric ulcer G4: 4%
Yoon [8] (2013)	Retro	93	No	2 (1.0–6.0)	A, B	45/3 (30–60/3–4)	95 (1Y) 92 (3Y)	86 (1Y) 54 (3Y)	RILD ≥ G2: 18% (≥ G3: 7%) Worsening of CP score ≥ 2: 10%	No bleeding or perforation
Sanuki [7] (2014)	Retro	185	NA	CP-A: 2.4 (0.5–5.0) CP-B: 2.7 (1.0–5.0)	A, B	CP-A: 40/5 CP-B: 35/5	99 (1Y)91 (3Y)	95 (1Y) 70 (3Y)	Worsening of CP score ≥ 2: 10% G5 hepatic failure: 1%	NA
Huertas [21] (2015)	Retro	77	NA	2.4 (0.7–6.3)	A, B8	45/3	99 (1Y) 97 (3Y)	99 (1Y) 84 (3Y)	RILD: 5%	Gastric ulcer G3: 1%, G4: 1% Colic ulcer G2: 1%
Katz [25] (2011)	Retro	18	No	4.0 (1.2–6.5)	A, B, C	50/10	9% of patients developed a new lesion within 12 months after transplantation	100% after LT and/or hepatic resection (median follow up 19.6 months)	Elevated liver enzyme G3: 5.6%	No GI toxicity ≥ G3
Bae [24] (2012)	Retro	20	No	< 5 cm (≤ 3 cm: 80%, > 3 cm: 20%)	A,B	50/10	85 (2Y)	88 (2Y)	No RILD ≥ G3	GI toxicity G1: 30% No GI toxicity ≥ G3
Present study	Retro	77	No	2.4 (0.8–5.6)	A, B	50/10 (35–50/10)	80 (3Y) 73 (5Y)	52 (3Y) 41 (5Y)	Worsening of CP score ≥ 2: 5.2%	GI bleeding G3: 1.3%

Abbreviations: *CP* Child-Pugh, *G* grade, *GI* gastrointestinal, *HCC* hepatocellular carcinoma, *LC* local control, *NA* not available, *OS* overall survival, *PVTT* portal vein tumor thrombus, Retro, retrospective, *RILD* radiation-induced liver disease

Iwata et al. assessed the outcomes of hypofractionated radiotherapy (50 or 55 Gy in 10 fractions) for HCC (*n* = 6) and liver metastasis (*n* = 12) and reported the 1-year local control rate for HCC as 100% without any gastrointestinal toxicity based on the dose constraints for the stomach, duodenum, and colon as 40 Gy in 10 fractions for ≤10 cc [23]. Likewise, Bae et al. reported the results of 50 Gy radiotherapy in 10 fractions as a salvage treatment for recurrent small HCC [24], although this study had limited experience due to enrollment of only patients with HCC not adjacent to the stomach or duodenum, no patient developed grade ≥ 3 toxicity with the 2-year local control rate of 85%. In addition, Katz et al. used a median total dose of 50 Gy in 10 fractions of radiotherapy and reported an excellent in-field control with minimal side effects as a bridge to transplantation for HCC [25]. Compared with all other previous studies mentioned earlier, our study discusses two issues. First, all the previous studies enrolled small study cohorts and did not present the long-term local control rate or treatment-related toxicity because the follow-up time after radiotherapy was relatively short. However, our study provided the long-term local control rate (72.6% at 5 years) with a low incidence (1.3%) of gastrointestinal complication as mentioned earlier. Second, in this study, the primary indication of this fractionation scheme is the HCC located < 2 cm of the gastrointestinal organs; the prescription dose ranged 35–50 Gy in 10 fractions depending on the dose constraints for normal organs in comparison to previous studies mentioned earlier.

As hypofractionated radiotherapy was performed for patients with recurrent HCCs after various sessions of previous treatments in this study, the 5-year OS rate was relatively low (40.9%) compared with the outcomes of surgical resection or RFA in patients with early-stage HCC. Multivariate analysis revealed that the number of previous treatment sessions was a significant prognosticator for the OS (HR = 1.08; 95% CI, 1.01–1.16; *P* = 0.029). Bae et al. reported that patients receiving fewer than two courses of local treatment exhibited a trend toward superior intrahepatic control compared with patients who had received more than two courses of local treatments [24]. Although they did not compare the OS based on the number of previous treatments, frequent intrahepatic recurrences may affect the survival outcomes in patients with recurrent HCC.

This study has some limitations. First, being a retrospective study, it might encompass a few potential risks, including selection bias and missing data. Second, in our patient cohort, there was heterogeneity in the treatment before and after radiotherapy, owing to the frequent incidence of recurrence, which could be associated with the overall patient’s outcomes. Nevertheless, this study enrolled a relatively large number of patients with HCC located adjacent to gastrointestinal tracts and also presented the long-term follow-up clinical outcomes.

## Conclusions

The 2-week schedule of hypofractionated radiotherapy is safe and effective, although the local control rate was not as high as that reported in recent studies on SBRT using 3–5 fractions. Overall, this radiotherapy scheme can be a potential alternative treatment option in patients with HCC located in the vicinity of the radiosensitive gastrointestinal tract when short-course SBRT is not feasible.

## Additional files

Additional file 1: Raw data of the patients. Patient characteristics and follow-up data exists. (XLSX 16 kb)

Additional file 2: Raw data of the treated lesions. Lesion characteristics and follow-up data exists. (XLSX 15 kb)

Additional file 3: **Table S1.** Factors associated with the local tumor control after hypofractionated radiotherapy. (DOCX 19 kb)

## Abbreviations

4D: 4-dimensional
CI: Confidence interval
CT: Computed tomography
GTV: Gross tumor volume
HCC: Hepatocellular carcinoma
HR: Hazard ratio
IGRT: Image-guided radiation therapy
ITV: Internal target volume
MRI: Magnetic resonance image
OS: Overall survival
PFS: Progression-free survival
PTV: Planning target volume
RECIST: Response Evaluation Criteria in Solid Tumors
RFA: Radiofrequency ablation
SBRT: Stereotactic body radiation therapy
TACE: Transarterial chemoembolization
