# Supplementary material for: Long-term outcomes of the 2-week schedule of hypofractionated radiotherapy for recurrent hepatocellular carcinoma
Source: BMC Cancer. 2018 Oct 26;18:1040. doi: 10.1186/s12885-018-4953-x (PMC6203968; doi:10.1186/s12885-018-4953-x)
Supplement: Supplementary file 3 — Table S1. Factors associated with the local tumor control after hypofractionated radiotherapy. (DOCX 19 kb) [file 12885_2018_4953_MOESM3_ESM.docx]

**Supplementary material**

**Additional file 3: Table S1.** Factors associated with the local tumor control after hypofractionated radiotherapy

|  | Univariate analysis | | |
| --- | --- | --- | --- |
| Variables | HR | 95% CI | *P* value |
| Sex | 1.84 | 0.67–5.07 | 0.241 |
| Age | 1.02 | 0.97–1.08 | 0.377 |
| Tumor size | 1.23 | 0.79–1.93 | 0.363 |
| Alpha-fetoprotein (log_10_) | 0.93 | 0.52–1.67 | 0.815 |
| Child-Pugh class | 0.70 | 0.20–2.51 | 0.586 |
| Number of previous treatment sessions | 1.03 | 0.89–1.18 | 0.717 |
| Total dose | 0.96 | 0.88–1.04 | 0.325 |

HR, hazard ratio; CI, confidence interval.
